# A C2H2-Type Zinc-Finger Protein from *Millettia pinnata*, MpZFP1, Enhances Salt Tolerance in Transgenic Arabidopsis

**DOI:** 10.3390/ijms221910832

**Published:** 2021-10-07

**Authors:** Zhonghua Yu, Hao Yan, Ling Liang, Yi Zhang, Heng Yang, Wei Li, Jaehyuck Choi, Jianzi Huang, Shulin Deng

**Affiliations:** 1 Guangdong Provincial Key Laboratory of Applied Botany, South China Botanical Garden, Chinese Academy of Sciences, Guangzhou 510650, China; yuee.zhonghua2000@gmail.com (Z.Y.); liangling20@mails.ucas.ac.cn (L.L.); yizhang@scbg.ac.cn (Y.Z.); hengy@scbg.ac.cn (H.Y.); 2 Department of Landscape Architecture, PaiChai University, Deajeon 35345, Korea; liwei7137@gmail.com (W.L.); jhchoi@pcu.ac.kr (J.C.); 3 Guangdong Provincial Key Laboratory for Plant Epigenetics, College of Life Sciences and Oceanography, Shenzhen University, Shenzhen 518060, China; yanhaonihao@sina.com; 4 College of Life Sciences, University of Chinese Academy of Sciences, Beijing 100049, China; 5 School of Biological and Food Processing Engineering, Huanghuai University, Zhumadian 463000, China; 6 Xiaoliang Research Station for Tropical Coastal Ecosystems, South China Botanical Garden, Chinese Academy of Sciences, Guangzhou 510650, China; 7 National Engineering Research Center of Navel Orange, Gannan Normal University, Ganzhou 341000, China

**Keywords:** C2H2 zinc finger protein, heterologous expression, *Millettia pinnata*, salt tolerance

## Abstract

C2H2 zinc finger proteins (ZFPs) play important roles in plant development and response to abiotic stresses, and have been studied extensively. However, there are few studies on ZFPs in mangroves and mangrove associates, which represent a unique plant community with robust stress tolerance. *MpZFP1*, which is highly induced by salt stress in the mangrove associate *Millettia pinnata*, was cloned and functionally characterized in this study. MpZFP1 protein contains two zinc finger domains with conserved QALGGH motifs and targets to the nucleus. The heterologous expression of *MpZFP1* in Arabidopsis increased the seeds’ germination rate, seedling survival rate, and biomass accumulation under salt stress. The transgenic plants also increased the expression of stress-responsive genes, including *RD22* and *RD29A*, and reduced the accumulation of reactive oxygen species (ROS). These results indicate that MpZFP1 is a positive regulator of plant responses to salt stress due to its activation of gene expression and efficient scavenging of ROS.

## 1. Introduction

Arable lands are suffering from continuous salinization at an annual rate of ~10% due to environmental changes and poor cultural practices. Approximately 50% of the total cultivated land area was predicted to be salinized by the year 2050 worldwide [1]. Salt stress has multiple deleterious effects on plant growth and is a major environmental factor reducing crop productivity. Thus, improving the salt tolerance of crops through genetic modification is a potential approach for optimum economic sustainability [1,2]. The generation of salt-resistant crops relies on the discovery of plant stress-responsive mechanisms and the availability of genetic resources.

Pongamia (*Millettia pinnata* syn. *Pongamia pinnata*) belongs to the semi-mangrove (or mangrove associate) growing within intertidal zones in tropical and subtropical regions, and possesses a high degree of salt tolerance [3,4]. Unlike true mangroves, Pongamia does not feature the salty glands or other specialized morphological traits required to endure salinity stress. It is suggested that the mechanisms that Pongamia uses to cope with the high saline environment are tightly linked to gene regulation and protein function [3,4,5]. Therefore, investigating the molecular mechanisms of Pongamia’s adaptation to saline environments offers promising insight into stress-tolerant crop breeding, and the salt tolerance genes derived from Pongamia may be highly efficient in the genetic modification of crops. The physiological mechanisms through which Pongamia responds to salt stress have been extensively studied [3,4,5,6]. These reports indicated that the hydrophobic cell-wall barriers and vacuolar sequestration of Na^+^ were among the key mechanisms conferring salt tolerance in Pongamia, and that osmolytes (myo-inositol and mannitol) and phytohormone (zeatin and jasmonic acid) were increased in salt-treated Pongamia [5,6]. Nevertheless, these analyses of Pongamia’s transcriptional regulation mechanisms are still insufficient for understanding its molecular responses to salinity stress. Transcriptome profiles of leaf and/or root tissues of Pongamia have been conducted to address the issue [7,8], while the detailed functional study of salt stress responsive gene is scarce. To date, only two salt-tolerant Pongamia genes, *MpCHI* and *MpCML40*, have been cloned and characterized [9,10]. The heterologous expression of *MpCHI* in yeast (*Saccharomyces cerevisiae*), which encodes the chalcone isomerase involved in flavonoid biosynthesis, could enhance the salt tolerance capacity of recipient cells [9]. Recently, we characterized a salt-induced Calmodulin-like gene, *MpCML40*, that enabled transgenic yeast and Arabidopsis to become salt-tolerant. The transgenic *MpCML40* Arabidopsis did not show any growth retardation, which differed from most genetically modified stress-tolerant plants [10].

Through transcriptional regulation, the complex network of plant development and abiotic stress responses is orchestrated by transcription factors (TFs), such as Zinc finger proteins (ZFPs) [11,12,13]. TFs bind to specific sequences in the promoters of their target genes, thereby regulating gene expression and affecting biological phenotypes. ZFPs form one of the largest TF families in plants and have been sub-classified into nine major types, C2H2, C2HC, C3H, C4, C6, C2HC5, C3HC4, C4HC3, and C8, according to the number and order of the cysteine and histidine residues, which bind tetrahedrally to zinc ions [11,14]. C2H2 ZFPs, also called classical or TFIIIA-type fingers, are one of the best-characterized DNA-binding motifs found in plant transcription factors, typically containing one to four conserved QALGGH motifs in zinc finger helices [11,14,15]. The Arabidopsis genome contains 176 C2H2-type ZFP genes and can be divided into three clades: A, B, and C. Clade C is further split into three sub-clades, C1, C2, and C3, based on sequence divergence [15,16]. The first C2H2-type ZFP gene discovered in plants was *EPF1*, from Petunia [12]. EPF1-related proteins have been demonstrated to enhance tolerance to abiotic stresses in several plant species [17,18,19,20].

In the past two decades, many C2H2 ZFP genes have been identified and studied in model plants, such as Arabidopsis, rice (*Oryza sativa*), soybean (*Glycine max*), and tomato (*Solanum lycopersicum*) [17,18,20,21,22]. The overexpression of Arabidopsis C2H2 zinc finger protein STZ/ZAT10 enhanced tolerance to salinity, heat and osmotic stress in transgenic Arabidopsis [18,23]. Another Arabidopsis C2H2 zinc finger protein, ZAT12, has been reported to be involved in oxidative, osmotic, salinity, high light, heat, and cold stress response [24,25]. The overexpression of rice C2H2 zinc finger protein genes, *ZFP179*, *ZFP182*, *ZFP245*, and *ZFP252*, has been shown to participate in salt, drought, and cold stress response [26,27,28,29]. The heterologous expression of the soybean C2H2 zinc finger gene *SCOF-1* has been shown to enhance low temperature stress tolerance in Arabidopsis and sweet potato [30,31]. Another soybean C2H2, GmZAT4, enhanced PEG and NaCl stress tolerance in transgenic Arabidopsis [32]. *GsZFP1*, a C2H2 zinc finger protein gene from wild soybean (*Glycine soja*), was induced by ABA and abiotic stress treatments. Transgenic *GsZFP1* Arabidopsis plants showed increased tolerance to cold and drought stresses by activating cold stress-responsive genes and ABA biosynthesis-related genes [33,34]. Transgenic alfalfa (*Medicago sativa* L.) expressing *GsZFP1* showed enhanced tolerance to cold and salt stresses [35]. In tomatoes, C2H2 zinc finger proteins have been reported to regulate trichome formation [36], ascorbic acid synthesis and salt tolerance [37], and mating system transition [38]. The heterologous expression of two C2H2 zinc finger protein genes, *CgZFP1* from chrysanthemum [39] and *IbZFP1* from sweet potato [19], improved salinity and drought tolerance in Arabidopsis. Some C2H2 ZFPs function in both biotic stress response and abiotic stress response. *CaZFP1*, a pathogen-induced pepper C2H2 zinc finger gene, endowed the transgenic Arabidopsis with drought tolerance and resistance against infection by *Pseudomonas syringae* [40]. Most of the available information suggests that ZFP genes from crops or other plant species are highly important for response to abiotic stresses, while few reports are available for mangroves or mangrove associates.

In the present study, we identified a ZFP gene, *MpZFP1*, from Pongamia. MpZFP1 belongs to the C1-2i subclass of C2H2 ZFPs containing two highly conserved QALGGH motifs. Under salt treatment, the *MpZFP1* gene was highly induced in roots. The heterologous expression of *MpZFP1* in Arabidopsis strongly enhanced the salt tolerance of transgenic plants, probably through efficient ROS scavenging.

## 2. Results

### 2.1. Cloning and Sequence Analysis of MpZFP1

**Figure 1:**
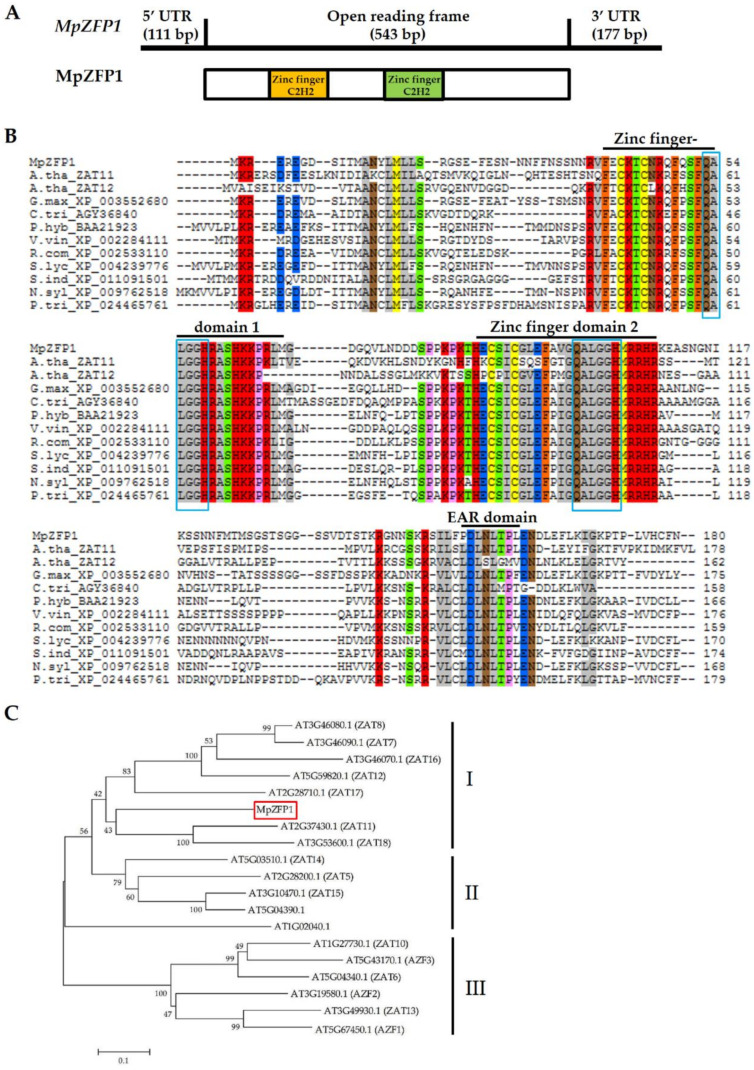
Analysis of the *MpZFP1* gene from Pongamia. (**A**) Structure of *MpZFP1* gene (upper panel) and MpZFP1 protein (lower panel). (**B**) Sequence alignment of MpZFP1 protein with its homologs from other species. The conserved zinc finger domains and EAR motif were marked and the two QALGGH consensus sequences are highlighted with a blue frame. Sequences of Glycine max (XP_003552680.1), Citrus trifoliata (AGY36840), Solanum lycopersicum (XP_004239776.1), Petunia X hybirda (BAA21923.1), Vitis vinifera (XP_002284111.1), Ricinus communis (XP_002528469.1), Nicotiana sylvestris (XP_009762518.1), Populus trichocarpa (XP_024465761.1), Sesamum indicum (XP_011091501.1), and Arabidopsis (AT2G37430.1/ZAT11, AT5G59820.1/ZAT12) were downloaded from GenBank. (**C**) Phylogenetic tree of MpZFP1 with Arabidopsis C1-2i clade C2H2 proteins. MpZFP1 is marked by a red box.

The full-length cDNA of the *MpZFP1* gene was cloned by 5′ and 3′ rapid amplification of cDNA ends (RACE) assays with four gene specific primers, based on the sequence of an EST from our previous study [7]. The full-length sequence of cDNA, which was deposited in GenBank under accession number MZ934391, comprised a 543 bp open reading frame (ORF), a 111 bp 5′ untranslated region (UTR), and a 177 bp 3′ UTR (Figure 1A). The corresponding protein contained 180 amino acids, and conserve domain analysis showed that *MpZFP1* contained two C2H2 zinc finger motifs (Figure 1A). A BLASTP homolog search of GenBank indicated that the deduced *MpZFP1* sequences showed high similarity with the predicted ZFPs of *Glycine max* (XP_003552680.1 67%), *Solanum lycopersicum* (XP_004239776.1, 57%), *Petunia X hybirda* (BAA21923.1, 55%), *Vitis vinifera* (XP_002284111.1, 55%), *Ricinus communis* (XP_002528469.1, 53%), and Arabidopsis (AT2G37430.1/ZAT11, 43%) (Figure 1B). All of these proteins contained two C2H2 zinc finger domains with QALGGH-conserved motifs and a short hydrophobic region consisting of core DLNL sequence, DLN-box. Apart from MpZFP1, in which the first Leu mutated to Pro, other proteins contained a consensus LXLXLX EAR motif (Figure 1B). The Arabidopsis ZAT11 belongs to the C1-2i subclade, which includes 20 members [15]. To classify the possible Arabidopsis ortholog of MpZFP1, a phylogenetic tree based on the amino acid sequences of MpZFP1 and the Arabidopsis C1-2i subclass proteins was constructed. The architecture of the phylogenetic trees suggested that the Arabidopsis C1-2i could be divided into three subsets, ZAT7-8/ZAT11-12/ZAT16-18, together with MpZFP1 merged into subset I (Figure 1C).

### 2.2. MpZFP1 Localizes in the Nucleus

**Figure 2:**
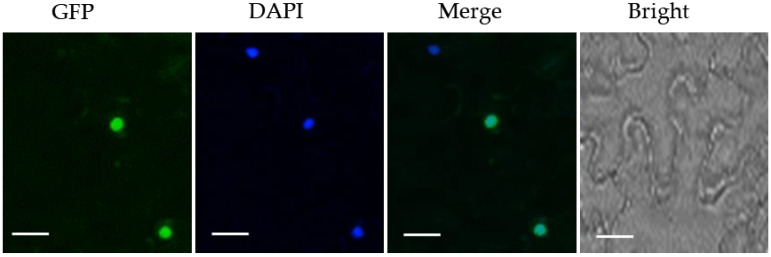
Subcellular localization of *MpZFP1-GFP*. Subcellular localization of *MpZFP1*-GFP was assayed with DAPI in tobacco leaf epidermal cells. The fluorescence signals were detected 48 h after infiltration. Bar = 50 μM.

Classic transcription factors locate in the nucleus and bind to specific DNA sequences to modulate gene expression. To address the subcellular localization of MpZFP1 protein, the ORF of *MpZFP1* was cloned and then inserted into the pCAMBIA-1302 expressing vector containing the green fluorescent protein gene (GFP) under the control of the CaMV35S promoter. The MpZFP1-GFP fusion protein was expressed in tobacco (*Nicotiana benthamiana*) leaves by the Agrobacterium tumefaciens-mediated transient expression system. The leaves were stained with DAPI ((4′,6-diamidino-2-phenylindole), which binds to double-stranded DNA as a nucleus marker, two hours before fluorescence microscopy detection. The results showed that the MpZFP1-GFP was co-localized with the DAPI signals (Figure 2). These results suggested that *MpZFP1* localized in the nucleus.

### 2.3. Expression of MpZFP Is Induced by NaCl

**Figure 3:**
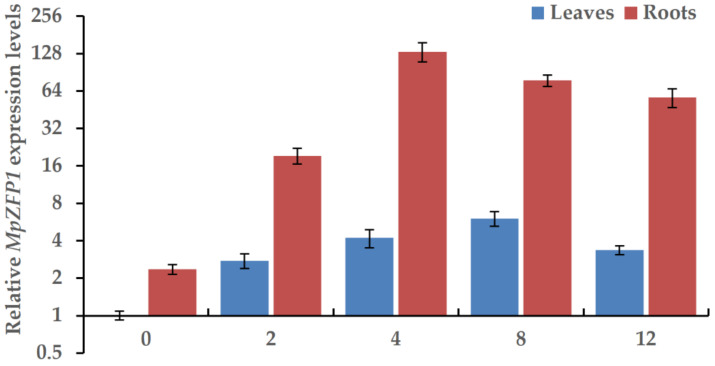
Relative expression levels of *MpZFP1* in Pongamia seedlings under salt stress. Relative expression levels of *MpZFP1* gene in leaves and roots after 500 mM NaCl treatment for 0, 2, 4, 8, 12 h was analyzed by quantitative RT-PCR. *MpActin* and *Mp18S* genes were used as internal references. All data were normalized to 0 h of leaves. Error bars show mean values (±SD) of three independent samples.

The previous transcriptome study indicated that *MpZFP1* transcripts accumulated in the salt-treated Pongamia cDNA library [7]. To investigate the dynamic changes of the gene expression during the early stage of salt stress in detail, we treated the Pongamia seedlings with a 500 mM salt solution for 0 h, 2 h, 4 h, 8 h, and 12 h. All the samples were collected at the same time, and the RNAs from the leaves and roots were extracted separately. Quantitative RT-PCR analysis showed that the expression of *MpZFP1* was significantly induced by NaCl. The expression of *MpZFP1* was higher in the roots compared to the leaves, and it reached its highest level (~56 folds compared to no stress) at 4 h of NaCl stress, followed by a decrease in the roots (Figure 3). Comparatively, the expression peaked at 8 h of NaCl stress in the leaves (~6 folds compared to no stress), and the induction levels were much lower in the leaves than in the roots (Figure 3). These results indicated that the MpZFP1 mainly functioned in the roots, especially under salt stress.

### 2.4. Heterologous Expression of MpZFP1 Strongly Enhances Salt Tolerance in Arabidopsis

**Figure 4:**
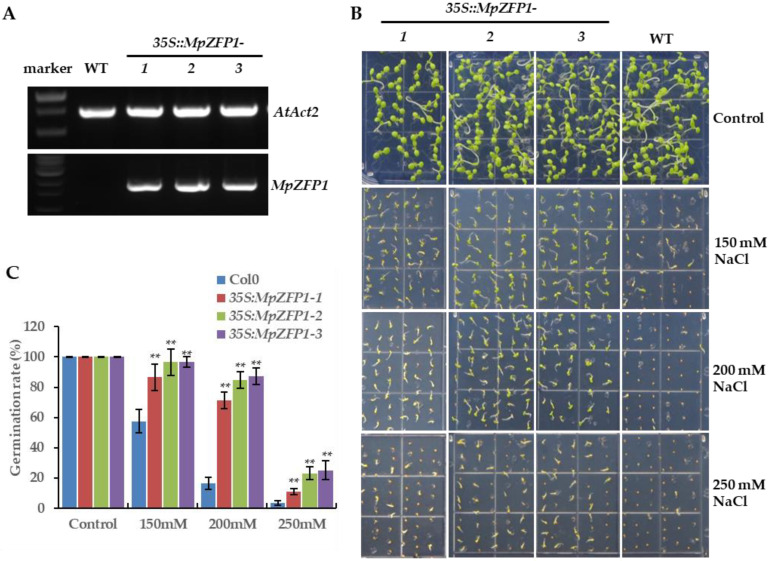
Ectopic expression of *MpZFP1* in Arabidopsis increases the seed germination of transgenic plants under salt stress. (**A**) Expression levels of *MpZFP1* in three independent *MpZFP1* heterologous expression lines. *AtAct2* was used as a control. (**B**) Typical phenotype of wild type and transgenic Arabidopsis seeds germinated on ½ MS medium contain 0 (Control), 150, 200, or 250 mM NaCl. (**C**) Quantification of (**B**), data shown are mean values of at least 50 individuals ± SD. ** *p* < 0.01 (Student’s *t*-test).

First, we generated the transgenic Arabidopsis plants carrying *MpZFP1* expressed from a CaMV (Cauliflower Mosaic Virus)-35S promoter to investigate possible functions of *MpZFP1* in salt stress response. The transgenic plants were generated by the method of Agrobacterium-mediated transformation [41]. Three genetically stable transgenic lines were selected for further experiments, and the significantly high expression of *MpZFP1* was confirmed by RT-PCR (Figure 4A). No obvious difference in development was observed in transgenic plants compared to wild-type plants under normal conditions. We checked the germination rate of the seeds for the *35S::MpZFP1* transgenic and wild-type Arabidopsis under salt stress. High concentrations of NaCl strongly inhibited seed germination in the wild-type plants. The germination rate of the wild-type Arabidopsis reduced to around 60%, 16%, and 4% under 150 mM, 200 mM, and 250 mM salt stress, respectively (Figure 4B,C). The 150 mM of NaCl had no obvious effects on seed germination in the transgenic plants; more than 90% of seeds were able to germinate under this condition (Figure 4B,C). The germination rate of the three transgenic plants varied from 70% to 87% under 200 mM of NaCl (Figure 4B,C). Moreover, the seeds of the transgenic line 2 and 3 plants showed a germination rate of more than 20% on the medium containing 250 mM of NaCl (Figure 4B,C).

**Figure 5:**
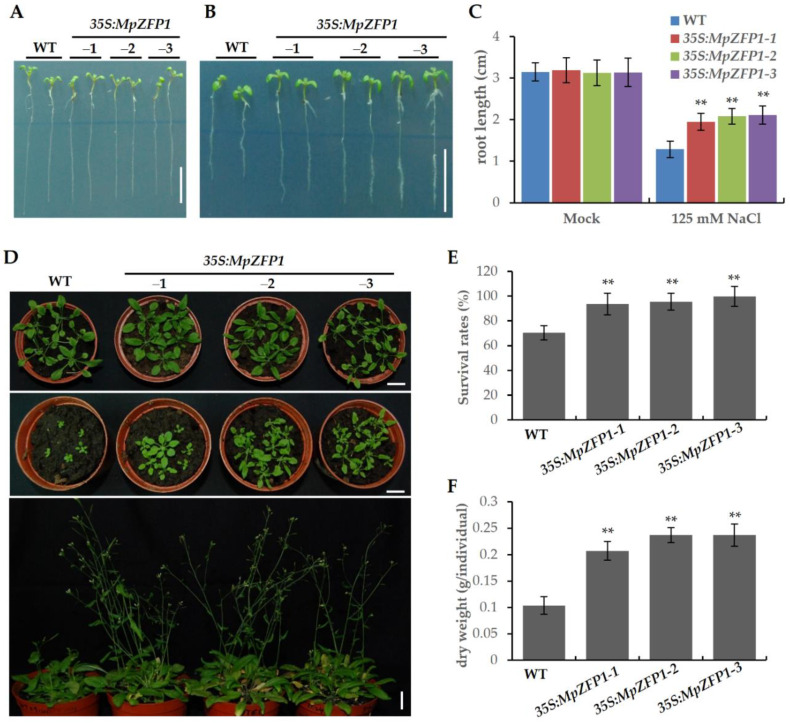
Ectopic expression of *MpZFP1* in Arabidopsis increases the salt tolerance of transgenic plants. Typical root length phenotype of 10-day wild-type and *MpZFP1* heterologous-expression Arabidopsis seedling grown on ½ MS medium (**A**) or ½ MS medium with 125 mM NaCl (**B**), bar = 1 cm. (**C**) Quantification of (**A**,**B**). Root length for the seedlings grown on normal (Mock) or 125 mM NaCl medium were measured. (**D**) Growth phenotype of 10-day recovery from non-treatment (upper panel) or 125 mM NaCl treated (middle panel) and growth phenotype of 30-day recovery from salt treatment (lower panel). (**E**) Survival rates of wild-type (WT) and *MpZFP1* transgenic plants after 10-day recovery from salt stress. (**F**) Dry weight of survived plants that recovered after 30 days. Data shown are mean values of at least 50 individuals ± SD (**C**,**E**,**F**). ** *p* < 0.01 (Student’s *t*-test).

Secondly, we measured the root lengths of the wild-type and transgenic seedlings under salt stress. The seedlings were cultured for three days on a normal ½ MS agar medium and then transferred to the medium supplemented with 0 or 125 mM NaCl. After one week of treatments, the root lengths did not show differences between the wild-type and transgenic seedlings in the medium without additional NaCl (Figure 5A,C). Under treatment with 125 mM NaCl, the root growth of all the plants was inhibited. However, the roots of the transgenic seedlings were significantly longer than those of the wild-type seedlings (Figure 5B,C).

Lastly, we monitored the growth phenotype of the *MpZFP1* transgenic plants under salt stress. We germinated and cultured the seedlings on a normal ½ MS agar medium for five days and then transferred them to the medium supplemented with 0 or 125 mM NaCl. The seedlings were then transferred to pots with soil after ten days of treatment. For the plants without salt stress, the transgenic lines grew as well as the wild type after ten days growth in soil (Figure 5D, upper panel). However, the transgenic plants performed much better in both survival rate and growth vigor after ten days of recovery (Figure 5D (middle panel) and Figure 5E). Almost all the transgenic plants survived the stress, but ~30% of the wild-type plants were killed by ten days of salt treatment (Figure 5E). All the surviving transgenic plants flowered and had seed sets after 30 days of recovery, but the wild-type plants were still in vegetative growth or had just started to flower (Figure 5D, lower panel). The transgenic plants had significantly greater biomass compared to the wild-type plants after 30 days of recovery from salt stress (Figure 5F).

Collectively, the results indicated that the *MpZFP1* heterologous expression endowed the transgenic plants with salt tolerance.

### 2.5. Heterologous Expression of MpZFP1 in Arabidopsis Enhances the Expression of Stress-Responsive Genes

**Figure 6:**
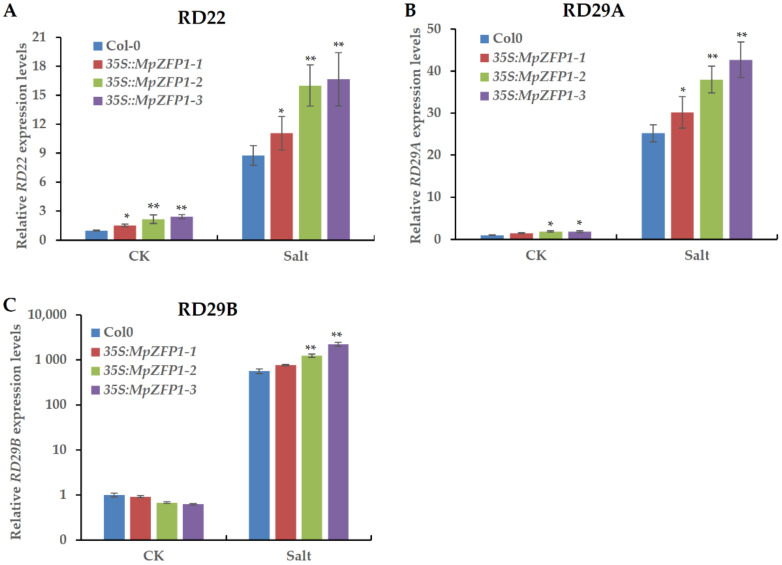
Expression levels of stress-responsive genes in wild type and 35S:*MpZFP1* transgenic plants. The expression levels of *RD22* (**A**), *RD29A* (**B**), and *RD29B* (**C**) of two-week-old Arabidopsis plants with (Salt) or without (CK) 200 mM NaCl treatment for three hours. *AtACT2* (*ACTIN2*) was used as a control. Mean values and standard deviations of three biological replicates are shown. * *p* < 0.05, ** *p* < 0.01 (Student’s *t*-test).

Transcriptional reprogramming is a major mechanism through which plants respond to stress, and the induction of stress-responsive genes is a hallmark of stress acclimation in plants. To investigate whether the ectopic expression of *MpZFP1* activated stress marker genes in the transgenic plants, we quantified the expression levels of three well-studied abiotic stress responsive genes, *RD22*, *RD29A*, and *RD29B*, in the transgenic plants with or without salt stress by quantitative RT-PCR. The expression levels of *RD22* and *RD29A* were significantly higher in the transgenic plants in both normal growth conditions and stress conditions (Figure 6A,B). The expression levels of RD29B were slightly lower in the transgenic plants under normal growth conditions but were higher under salt stress than in the wild-type plants (Figure 6C). Generally, the expression levels of the three stress-responsive genes were activated to higher levels by the expression of *MpZFP1*, especially under salt stress.

### 2.6. Heterologous Expression of MpZFP1 in Arabidopsis Improves ROS Scavenging

**Figure 7:**
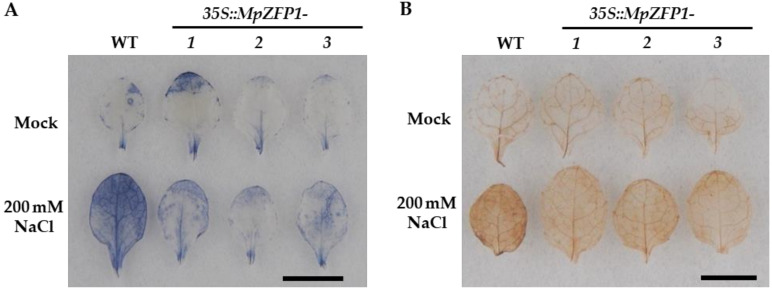
NBT and DAB staining of wild-type and 35S:*MpZFP1* transgenic Arabidopsis. The rosette leaves of three-week-old Arabidopsis were used for staining. The leaves were soaked in ½ MS liquid medium containing 0 mM (mock) or 200 mM of NaCl for 3 h and then transferred into NBT (**A**) or DAB (**B**) solution for staining. The experiments were repeated twice and at least 10 leaves for each genotype were assayed. The data obtained are shown here. Bar = 1 cm.

Abiotic stresses such as salt, drought, and high temperature usually lead to secondary oxidative stress resulting in reactive oxygen species (ROS) overaccumulation. Excess ROS cause damage to DNA, protein, and lipid membranes, which may ultimately lead to cell death. To analyze the ROS induced by salt stress, NBT (nitroblue tetrazolium) and DAB (3, 3′-diaminobenzidine) staining assays were performed to detect the contents of H_2_O_2_ and O_2_^−^ under mock or 200 mM-NaCl treatment. The leaves of the transgenic plants showed lighter staining colors than those of the wild-type plants did after salt treatment (Figure 7A,B), indicating lower contents of H_2_O_2_ and O_2_^−^ in the transgenic plants.

## 3. Discussion

Zinc finger proteins constitute one of the largest families that have been claimed to play critical roles in plant development and response to environmental stresses. The zinc finger domain can bind to DNA or RNA, and serve as a mediator of protein–protein interaction, which contributes to the functional diversity of this protein family [13,14,15,20]. Although C2H2 ZFPs have been extensively studied and found to be involved in responses to abiotic stresses, such as drought, salt, high temperatures, cold and high light [11,20,42], there have been few reports on this type of protein in mangroves or mangrove associates, which represent a unique plant community with high and robust stress tolerance.

We cloned and functionally characterized the first stress-responsive ZFPs, MpZFP1, from Pongamia, a typical mangrove associate. Phylogenetic analysis showed that the MpZFP1 belonged to the C1-2i type of C2H2, with the highest similarity with ZAT11/12 in Arabidopsis (Figure 1). As the other homolog in C1-2i, the MpZFP1 has two dispersed zinc finger domains. The other motif that embodies this family is a short hydrophobic region consisting of the core LXLXL sequence, the EAR motif, at the C-terminus [15,18]. The EAR domain, the smallest known repressive domain, was initially found in APETALA2 (AP2)/ETHYLENE RESPONSE FACTOR (ERF) proteins [43]. Many plant C1-2i C2H2 ZFPs containing the EAR motif have also been shown to function as repressors, such as AZF1/2/3, ZAT7, and ZAT10/11/12 in Arabidopsis [15,18,43]. The first Leu mutated to Pro residue in the core LXLXL motif in MpZFP1 (Figure 1B), which brought uncertainty to the issue of whether MpZFP1 functioned as a transcription repressor in the same way as its close homolog ZAT12. Through microarray study, many cold positive regulators were found to be suppressed by ZAT12, which was consistent with its function as a repressor [25]. However, ZAT12 played positive roles in cold tolerance, with its full function yet to be uncovered.

Since the transcripts of *MpZFP1* were highly induced by salt stress (Figure 2), we focused on its function in salt stress response. Arabidopsis transgenic plants harboring 35S:*MpZFP1* were generated and three independent lines varied in expression levels, marked as line 1 to 3 from, low to high (Figure 4A), were chosen for stress tolerance assays. We first checked the germination rate of *35S:MpZFP1* heterologous-expression plants under different salt concentrations. There was no difference between the wild type and the three lines of transgenic plants when germinated on the control medium without additional NaCl (Figure 4B,C). The germination rates of the transgenic lines were much higher than those of the wild type under salt stress, with line 3 showing the best performance, which was consistent with the expression levels of *MpZFP1* (Figure 4B,C). The inhibition of root growth under salt stress was alleviated by the expression of *MpZFP1* (Figure 5A–C). The transgenic plants also showed a better survival rate and biomass accumulation after salt stress (Figure 5D–F). These results indicate that the MpZFP1 is a positive regulator of salt stress tolerance. To gain insight into the molecular function of the MpZFP1, we examined the expression level of several stress-responsive genes in transgenic plants. The expression levels of *RD22* and *RD29A* were activated by MpZFP1 even under normal growth conditions (Figure 6A,B). A similar result was reported for the GsZFP1, a C2H2 protein from the wild soybean. The expression of *RD22* and *RD29A* was also activated by the heterologous expression of GsZFP1 in Arabidopsis [33,34]. Unlike *RD29A*, which is activated by the AP2/ERF transcription factor DREB, *RD29B* is a direct target of bZIP transcription factors, such as ABI5 and ABFs [44]. A slightly decreased expression level was detected for *RD29B* in *MpZFP1* transgenic plants (Figure 6C), probably through the inhibition of *ABFs* and *ABI5*, whose expression was suppressed by GsZFP1 in Arabidopsis [34].

Abiotic stresses always lead to the over-accumulation of ROS, which causes significant damage to the cells. The MpZFP1 close Arabidopsis homolog ZAT12 plays critical roles in reactive oxygen and abiotic stress signaling [24]. To investigate the possible roles of MpZFP1 in the regulation of ROS levels under salt stress, we used NBT and DAB staining to monitor the contents of H_2_O_2_ and O_2_^−^. The leaves of the *MpZFP1* transgenic plants showed much lighter staining colors under 200 mM-NaCl treatment, which indicated that MpZFP1 might enhance salt tolerance through efficient ROS scavenging. Taken together, these results indicated that the closely related C2H2 ZFPs from diverse plants functioned conservatively.

## 4. Materials and Methods

### 4.1. Plant Materials and Growth Conditions

The Arabidopsis thaliana ecotype Columbia (Col-0) was used as a wild type to generate *35S:MpZFP1* transgenic plants. The transgenic plants were generated by agrobacterium-mediated flora dipping [41]. The Arabidopsis seeds were germinated and cultivated on ½ MS agar plates (half MS basal salts supply with 1% sucrose and 0.8% agar) in a tissue culture room, with a long-day (16 h light and 8 h dark) photoperiod, at 22 °C. Ten-day-old seedlings were transferred into pots with soil and grown in a growth chamber, with a long-day photoperiod, at 22 °C. The Pongamia seeds were soaked in tap water at 28 °C in a growth cabinet until radicles appeared. These germinated seeds were then planted in soil for further growth.

### 4.2. Full-Length cDNA Cloning, Motif Prediction and Phylogenetic Analysis

SMARTer^TM^ RACE cDNA Amplification Kit (Takara Bio, Otsu, Japan) was used for 5′ and 3′ RACE. The putative *MpZFP1* fragment from the Pongamia transcriptome dataset was used as a template for designing gene-specific internal primers. A nested PCR protocol was carried out with the primers listed in Table 1. The 5′ and 3′ ends of cDNA were sequenced and assembled into full-length cDNA. The conserved motifs of MpZFP1 and homologous protein were analyzed using SMART (Simple Modular Architecture Research Tool) and SMS online tools [45]. MpZFP1 and 18 Arabidopsis C1-2i proteins (download from TAIR) were used for phylogenetic analysis. The phylogenetic tree was constructed using the Neighbor-Joint method implemented in the MEGA X program [46].

### 4.3. Subcellular Localization Analysis

The *MpZFP1-GFP* fusion expression vector was transferred into agrobacteria strain GV3101. The four-week-old *N. benthamiana* leaves were used for agrobacteria-mediated transient expression. GFP fluorescence was taken after 48 h of infiltration using a LEICA SP8 STED 3X fluorescence microscope confocal system. Two hours before microscope detection, the infiltrated leaves were stained with DAPI to mark the nucleus.

### 4.4. RNA Extraction and Quantitative Real-Time PCR

One-month-old Pongamia seedlings were transferred from soil into ½ MS liquid medium. After overnight culture, the normal ½ MS liquid medium was replaced by a ½ MS liquid medium containing 500 mM NaCl. The seedling samples were collected 0, 2, 4, 8, 12 h after treatments. Two-week-old Arabidopsis were used for stress-responsive gene analysis. The seedlings were transferred to a ½ MS liquid medium from a ½ MS agar plate one day before treatment. The samples were frozen by liquid nitrogen after 3 h treatment with or without 200 mM NaCl in a fresh ½ MS liquid medium. The total RNAs were isolated using TRIzol™ Plus (Takara Bio, Otsu, Japan) following the manufacturer’s protocol. About 1000 ng of total RNA were digested by DNase I for 30 min at 37 °C before reverse transcription. The DNase digestion was terminated by the addition of 25 mM EDTA and followed by incubation at 70 °C for 10 min. A first-strand cDNA synthesis was performed using an oligo(dT) 18 primer and GoScript™ Reverse Transcriptase (Promega, Madison, WI, USA). Subsequently, qRT-PCR was performed on a Roche LightCycler 480 with gene-specific primers and an SYBR Green mix (Takara Bio, Otsu, Japan). All primers used in the qRT-PCR are listed in Table 1.

### 4.5. Phenotype Analysis of Wild-Type and 35S:MpZFP1 Transgenic Arabidopsis Plants

For the germination rate assays, at least 100 seeds of wild-type and transgenic plants were sowed on a ½ MS medium containing different concentrations of NaCl (0, 150, 200, and 250 mM). After two days of stratification at 4 °C in the dark, the seeds were transferred to light for the assessment of their germination rates. A seed was considered as germinated when the radical protruded through its envelope.

For the root length assay, the seedlings were germinated and grown for three days on a normal ½ MS agar medium, and then transferred to the medium containing 0 or 125 mM of NaCl. The root length was measured by ImageJ software after 7 days of treatment. At least 30 plants for each genotype in each biological repetition were checked.

For the growth assay, the seedlings were germinated and grown for five days on a normal ½ MS agar medium, and then transferred to the medium containing 0 or 125 mM of NaCl. The seedlings were transferred to pots with soil after 10 days of treatment. The survival rate was scored after 10 days of recovery. To address the influence of salt stress on biomass, the 30-day-recovery seedlings were dried in a drying oven for three days at 85 °C and their weights were measured. At least 30 plants for each genotype in each biological repetition were checked.

### 4.6. DAB and NBT Staining

Three-week-old Arabidopsis leaves grown on a ½ MS agar plate were used for DAB staining and NBT staining. The leaves were vacuumed in a ½ MS liquid medium containing 200 mM of NaCl for 5 min and then soaked for another 4 h. Staining was performed by vacuuming the leaves in a 1 mg/mL DAB solution or a 0.2% NBT solution for 5 min and then staining for another 4 h.

## 5. Conclusions

C2H2 ZFPs have been reported to regulate responses to abiotic stress in a number of plants. We functionally characterized a nucleus-localized C2H2 ZFP, *MpZFP1*, which was involved in salt tolerance in Pongamia in the study. The ectopic expression of *MpZFP1* in Arabidopsis greatly enhanced the salt tolerance of transgenic plants through the activation of stress-responsive gene expression and ROS scavenging. Abiotic stresses, including salt stress, frequently impose constraints on plant distribution and growth performance, which threatens food security. The transgenic lines grew as well as the wild-type plants under normal growth conditions, which makes them ideal candidates for the breeding of stress-tolerant crops by using genetic modification.

## Figures and Tables

**Table 1:** Primers used in this study.

Name	Sequences (5′–3′)	Description
MpActin_F	AGAGCAGTTCTTCAGTTGAG	RT-PCR
MpActin_R	TCCTCCAATCCAGACACTAT	RT-PCR
Mp18s_RtF	GCTCGTAGTTGGACCTTG	RT-PCR
Mp18s_RtR	TTCGCAGTTGTTCGTCTT	RT-PCR
MpZFP1_RtF	TTTGCTGTAGGACAAGCTTTGGGA	RT-PCR
MpZFP1_RtR	CGGGAAACAAAATTGATCTCTTGCT	RT-PCR
RD22_RtF	ACGTCAGGGCTGTTTCCAC	RT-PCR
RD22_RtR	TACTTCTGTTTGTGACACACC	RT-PCR
RD29A_RtF	TTCCGTTGAAGAGTCTCCAC	RT-PCR
RD29A_RtR	AACAAAACACACATAAACATCC	RT-PCR
RD29B_RtF	CCACGGTCCGTTGAAGAGTC	RT-PCR
RD29B_RtR	CAAAAACACAAACATTCAAAAGC	RT-PCR
AtAct2_RtF	GACCTTTAACTCTCCCGCTATG	RT-PCR
AtAct2_RtR	GAGACACACCATCACCAGAAT	RT-PCR
Long-UPM	CTAATACGACTCACTATAGGGCAAGCAGTGGTATCAACGCA	RACE
Short-UPM	CTAATACGACTCACTATAGGGC	RACE
NUP	AAGCAGTGGTATCAACGCAGAGT	RACE
MpZFP1_5′GSP	TGGCTTCTTATGGCTTGCACGGT	RACE
MpZFP1_5′NGSP	TGGCGGTTACATGTCTTGCACTCGAAG	RACE
MpZFP1_3′GSP	GGACAAGCTTTGGGAGGCCACATGA	RACE
MpZFP1_BamHIF	TATGGATCCATGAAGAGAGAAAGGGAAGGT	clone
MpZFP1_SacIR	CACGAGCTCTCAATTGAAACAATGAACCAAAG	clone
